# Ferroptosis: a potential bridge linking gut microbiota and chronic kidney disease

**DOI:** 10.1038/s41420-024-02000-8

**Published:** 2024-05-15

**Authors:** Zi-Hui Mao, Zhong-Xiuzi Gao, Shao-Kang Pan, Dong-Wei Liu, Zhang-Suo Liu, Peng Wu

**Affiliations:** 1 Traditional Chinese Medicine Integrated Department of Nephrology, The First Affiliated Hospital of Zhengzhou University, Zhengzhou, PR China; 2 Institute of Nephrology, Zhengzhou University, Zhengzhou, PR China; 3 Henan Province Research Center for Kidney Disease, Zhengzhou, PR China; 4 Key Laboratory of Precision Diagnosis and Treatment for Chronic Kidney Disease in Henan Province, Zhengzhou, PR China

**Keywords:** Chronic kidney disease, Cell death

## Abstract

Ferroptosis is a novel form of lipid peroxidation-driven, iron-dependent programmed cell death. Various metabolic pathways, including those involved in lipid and iron metabolism, contribute to ferroptosis regulation. The gut microbiota not only supplies nutrients and energy to the host, but also plays a crucial role in immune modulation and metabolic balance. In this review, we explore the metabolic pathways associated with ferroptosis and the impact of the gut microbiota on host metabolism. We subsequently summarize recent studies on the influence and regulation of ferroptosis by the gut microbiota and discuss potential mechanisms through which the gut microbiota affects ferroptosis. Additionally, we conduct a bibliometric analysis of the relationship between the gut microbiota and ferroptosis in the context of chronic kidney disease. This analysis can provide new insights into the current research status and future of ferroptosis and the gut microbiota.

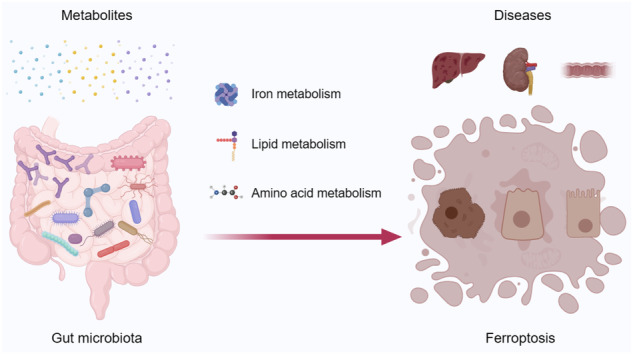

## Facts

1. Ferroptosis is a novel form of iron-dependent, lipid peroxidation-driven programmed cell death.
2. Ferroptosis is regulated by various metabolic pathways, particularly those involved in iron and lipid metabolism.
3. The gut microbiota plays a crucial role in maintaining the metabolic balance of the host.
4. The gut microbiota is associated with ferroptosis in various organs, tissues, and diseases.

## Open Questions

1. What potential connections exist between ferroptosis and the gut microbiota?
2. How does the gut microbiota regulate ferroptosis?
3. Can targeting the gut microbiota become a therapeutic strategy to alleviate ferroptosis-related diseases?

## Introduction

Ferroptosis is a distinct form of cell death characterized by iron-dependent, lipid peroxidation-driven, and accumulation of reactive oxygen species (ROS) [1]. This concept was initially proposed by Dr. Brent R Stockwell in 2012. Ferroptosis is directly influenced by the transport of cystine, the activity of glutathione peroxidase 4 (GPX4), the reduction of Fe, and the Fenton reaction. Cystine is transported across the membrane via system Xc^-^, where it is converted to cysteine and forms reduced glutathione (GSH) in conjunction with glutamic acid and glycine. The conversion of GSH to oxidized glutathione catalyzed by GPX4 coincides with a decrease in polyunsaturated fatty acid (PUFA)-O-OH. PUFA-O-OH then reacts with Fe^2+^ to generate ROS. GSH depletion, reduced GPX4 activity, and decreased cellular antioxidant capacity collectively lead to lipid peroxidation and ROS accumulation, ultimately resulting in ferroptosis. Therefore, ferroptosis is closely associated with the metabolism of lipids, iron, and mercaptans, and is triggered in cells with dysregulated redox metabolism [2].

The gut microbiota is a significant “organ” of the human body and plays a crucial role in human health. The microbiota that colonizes the mammalian gut gradually establishes a balanced symbiotic relationship during host development, resulting in a mutually beneficial correlation [3]. Disruption of the intestinal mucosal barrier, alterations in the microbiota, and microbial translocation lead to systemic inflammation, further impacting the host’s immune and metabolic homeostasis [4]. The gut microbiota influences the host’s inflammatory state and immune system, which in turn affects the prognosis of disease. Moreover, the intestinal microbiota contributes to the progression of host diseases through its metabolites, including short chain fatty acids (SCFAs), bile acids, tryptophan, branched-chain amino acids, and uremic toxins [5]. With the development of research, attention has gradually been given to whether the gut microbiota has the ability to regulate ferroptosis. The gut microbiota plays a regulatory role in ferroptosis through its microbial composition and metabolites, with different microbiota having varying effects on ferroptosis [6]. Pathogenic bacteria tend to promote ferroptosis and aggravate disease progression, while probiotics can prevent ferroptosis and alleviate disease progression [7]. However, it is important to note that the promotion or inhibition of ferroptosis may have different implications for tumor diseases and their progression. Hence, further exploration of the impact of the gut microbiota on ferroptosis will help us gain a deeper understanding of the mechanisms underlying disease occurrence and progression, as well as strategies to delay ferroptosis.

Chronic kidney disease (CKD) is a prevalent chronic progressive disease characterized by abnormal kidney structure and dysfunction resulting from various underlying conditions. The global prevalence of CKD is growing rapidly, leading to an increased risk of cardiovascular events, renal failure, and mortality [8]. As an organ responsible for metabolite excretion and reabsorption, the kidney is highly susceptible to disruptions in the redox balance. When intracellular iron accumulation and the redox system become imbalanced, excessive production of lipid peroxides will occur, ultimately inducing ferroptosis. Additionally, CKD is associated with lipid metabolism disorders and lipid accumulation. The buildup of lipids can activate the innate immune system, promote inflammatory fibrosis, trigger mitochondrial and kidney cell damage, and drive the progression of CKD [9]. Furthermore, the gut microbiota and its metabolites present intriguing therapeutic targets for delaying CKD progression and reducing uremic toxicity [10]. Evidently, the gut microbiota affects ferroptosis by regulating iron metabolism and related metabolites, and ferroptosis plays a crucial role in the progression of CKD. The progression of CKD leads to more severe dysbiosis, resulting in higher levels of ferroptosis, thus forming a vicious cycle and posing serious health risks. By comprehensively studying the relationship among ferroptosis, the gut microbiota, and CKD, new insights may be gained for effective personalized intervention strategies such as tailored diets and microbial interventions. This review explores the impact of the gut microbiota on ferroptosis and its underlying mechanisms from a metabolic perspective. In addition, we conduct a bibliometric analysis of studies investigating the relationship among ferroptosis, the gut microbiota, and CKD, aiming to provide a more comprehensive and in-depth understanding of the mechanisms of CKD progression and potential therapeutic approaches for associated complications.

## Key ferroptosis-related metabolic pathways

**Fig. 1:**
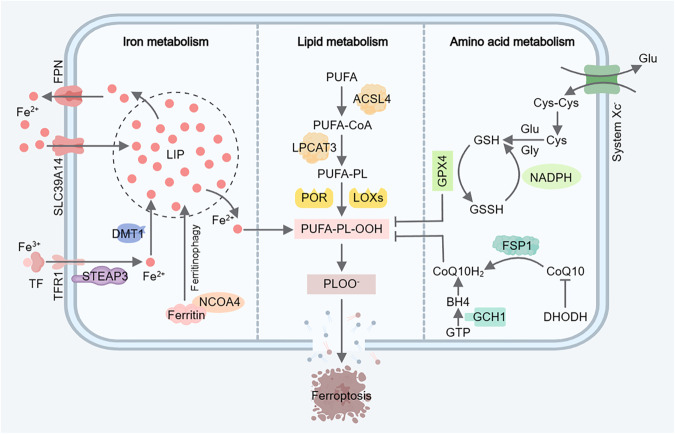
Key ferroptosis-related metabolic pathways. Ferroptosis is driven by iron-dependent lipid peroxidation, and ferroptosis sensitivity is intricately linked to iron metabolism, PUFA metabolism, and GSH synthesis. Iron metabolism encompasses processes such as iron absorption, transport, storage, and utilization. Lipid metabolism plays a crucial role in driving ferroptosis by regulating PUFA supply and PL synthesis, thereby activating the peroxidation of specific lipids that are incorporated into membrane lipids. The classical ferroptosis-suppressing pathway involves the uptake of Cys-Cys and the synthesis of GSH. GPX4 reduces PUFA peroxidation while simultaneously converting GSH to oxidized glutathione. FPN Ferroportin, TF Transferrin, TFR1 Transferrin receptor 1, LIP Labile iron pool, STEAP3 Six-Transmembrane Epithelial Antigen of Prostate 3, DMT1 Divalentmetal-iontransporter-1, NCOA4 Nuclear receptor coactivator 4, PUFA polyunsaturated fatty acid, ACSL4 Acyl- coenzyme A synthetase long-chain family 4, PUFA-CoA Coenzyme A-activated polyunsaturated fatty acid, LPCAT3 lysophosphatidylcholine acyltransferase 3, PUFA-PL polyunsaturated fatty acid-containing phospholipid, POR cytochrome P450 oxidoreductase, LOXs lipoxygenases, GSH reduced glutathione, GSSH oxidized glutathione, GPX4 glutathione peroxidase 4, Cys-Cys Cystine, Cys cysteine, Glu glutamate, Gly glycine, FSP1 ferroptosis suppressor protein 1, CoQ10 ubiquinone, CoQ10H_2_ ubiquinol, DHODH dihydroorotate dehydrogenase, GCH1 GTP cyclohydrolase 1, BH4 tetrahydrobiopterin.

In reviewing the significant advancements in ferroptosis over the past decade, Stockwell comprehensively summarized the mechanisms and biological importance of ferroptosis in the domains of cell metabolism, ROS, and iron regulation [11]. Ferroptosis sensitivity is closely linked to various biological processes, including amino acid metabolism, iron metabolism, PUFA metabolism, GSH synthesis, and phospholipid (PL) synthesis. The key points of ferroptosis regulation are cystine transport, fatty acid synthesis, and iron transport. Moreover, several metabolic pathways are involved in the regulation of ferroptosis. Therefore, it is necessary to elucidate the principal metabolic pathways associated with ferroptosis, such as lipid metabolism, iron metabolism, and amino acid metabolism pathways (Fig. 1).

### Lipid metabolism

Lipid metabolism plays a pivotal role in modulating ferroptosis by regulating PL peroxidation. While lipid peroxide substrates and oxidants that drive ferroptosis are generated during normal cell metabolism, only specific lipids that are activated and incorporated into membrane lipids ultimately induce ferroptosis [11]. Lipid peroxidation at the membrane can disrupt the ion balance and increase membrane permeabilization [12]. The composition of PLs is controlled by lipid metabolism through the regulation of fatty acid supply(especially PUFAs) and remodeling of PLs synthesis, which in turn affects cell susceptibility to ferroptosis [13]. Acyl-coenzyme A synthetase long-chain family (ACSL) 4 and lysophosphatidylcholine acyltransferase 3 are critical for ferroptosis because they promote the activation and incorporation of PUFAs into membrane lipids. The conversion of fatty acids to acyl-coenzyme A (CoA) esters is a crucial regulatory step in ferroptosis [14–16]. Remodeling lipid metabolism, particularly the redistribution of oxidizable PUFAs, leads to increased lipid peroxidation and induces ferroptosis in tumors [17]. Similarly, removal of the oxidized-PUFA tails on PLs can suppress ferroptosis [18]. In contrast to PUFAs, exogenous monounsaturated fatty acids effectively limit lipid peroxidation and block ferroptosis under ACSL3-dependent conditions [19].

In addition to oxidants that induce lipid peroxidation, inhibitors that prevent lipid peroxidation are also produced during normal metabolism. Among these inhibitors, GPX4 plays a central role in ferroptosis regulation, and GSH, which prevents oxidative stress caused by oxidants such as hydrogen peroxide, can restrain the accumulation of lipid ROS. The depletion of GSH increases the susceptibility of cells to ferroptosis, while GSH synthesis confers resistance to ferroptosis [20, 21]. Mechanisms that modulate the degradation of GPX4 also participate in regulating ferroptosis sensitivity. Both ferroptosis-inducer-56 and chaperone-mediated autophagy facilitate ferroptosis by inducing the degradation of GPX4 [22]. Additionally, there are other systems independent of GPX4 that regulate ferroptosis, such as ferroptosis suppressor protein 1 (FSP1)/ubiquinone (CoQ10) [23], dihydroorotate dehydrogenase (DHODH) [24], GTP cyclohydrolase-1 (GCH1)/tetrahydrobiopterin (BH4) [25], and amino acid oxidase interleukin-4-induced-1/indole-3-pyruvate [26]. Cholesterol, as a major component of the cell membrane, is involved in the synthesis of CoQ10 through an intermediate (isopentenyl pyrophosphate) in its synthesis pathway, thereby influencing ferroptosis. Furthermore, isopentenyl pyrophosphate can be utilized by a selenium protein to regulate GPX4 synthesis and increase ferroptosis sensitivity [27]. In summary, lipid peroxidation and disrupted lipid metabolism exacerbate ferroptosis, affecting disease progression and prognosis. A deeper understanding the mechanism of lipid metabolism in ferroptosis is of great significance for the development of new treatment strategies and preventive measures.

### Iron metabolism

Membrane lipid peroxidation with PUFA tails requires the involvement of the labile iron pool, which accelerates the Fenton reaction (Fe^2+^ + HOOH→Fe^3+^ + OH^−^ + OH^˙^), as well as iron-dependent enzymes such as lipoxygenase and cytochrome P450 oxidoreductase [28]. Therefore, iron metabolism is significantly important for the regulation of ferroptosis. Ferritin, an essential iron-storage protein, chelates free iron into Fe^3+^ to prevent the Fenton reaction. Transferrin and ferroportin (FPN) are responsible for iron transport. The cellular abundance of iron, and hence the sensitivity to ferroptosis, is determined by the availability of ferritin, the level of transferrin, and the function of FPN [29, 30]. Fe^3+^ is taken up into cells in the form of ferritin through transferrin and transferrin receptor 1 and is then reduced to bivalent ferritin by the six-transmembrane epithelial antigen of prostate 3. Subsequently, bivalent ferritin is deproteinized via divalent metal-ion transporter-1 or metal transporter ZIP14 (Slc39a14) to form Fe^2+^ and enters the labile iron pool. Excess Fe^2+^ is transported out of cells through FPN. The accumulation of Fe^2+^ leads to the Fenton reaction between Fe^2+^ and PUFAs-O-OH, which generates ROS, accelerates lipid peroxidation, and induces ferroptosis. Thus, the critical aspect of iron metabolism regulation in ferroptosis lies in controlling the capacity of the labile iron pool.

A recent study suggested that O-GlcNAcylation modulates ferroptosis by regulating the content of labile iron [31]. The transcription factor NUPR1 prevents ferroptosis by reducing the accumulation of labile iron and oxidative damage [32]. Mice fed a high-iron diet and mice with mutations associated with hereditary hemochromatosis (an iron overload disease caused by inherited mutations in genes that regulate iron metabolism) both developed ferroptosis-related liver damage [33]. Conversely, the ubiquitin ligase E3 HUWE1/MULE, which acts as a negative regulator of ferroptosis, regulates iron metabolism by targeting transferrin receptor 1 and counteracts abnormal iron accumulation, thereby alleviating acute liver injury caused by hepatic ischemia-reperfusion (I/R) [34]. Under hypoxic conditions, human macrophages inhibit ferritin autophagy by reducing the expression of nuclear receptor coactivator 4 (NCOA4), resulting in increased mitochondrial ferritin expression and decreased intracellular free iron, thus regulating ferroptosis [35]. Similarly, tripartite motif-containing protein 7 mediates ferritin autophagy and ferroptosis in human glioblastoma cells by binding to NCOA4 [36]. Additionally, NCOA4-ferritin heavy polypeptide 1-mediated iron metabolism disorder is related to retinal ganglion cell ferroptosis following pathologically high intraocular pressure injury [37]. A study indicated that histone deacetylase inhibitors, which are used clinically to treat certain cancers, can increase ferroptosis-induced cell death, possibly due to increased iron accumulation and decreased FPN expression after epithelial-to-mesenchymal transition mediated by histone deacetylase inhibitors [38]. These findings highlight a significant link between iron metabolism and susceptibility to ferroptosis.

### Amino acid metabolism

Ferroptosis is dependent on cystine transport and the synthesis of GSH. GSH, a significant antioxidant and enzyme cofactor in cells, consists of glutamic acid, cysteine, and glycine. In particular, cysteine is the rate-limiting substrate for GSH synthesis. Cysteine can be derived from cystine transported by System Xc^-^ or produced through endogenous transsulfuration [39]. Dixon et al. demonstrated that erastin depletes cysteine and GSH by blocking cystine uptake via System Xc-, leading to ferroptosis [40]. In the absence of cystine, certain tumor cells can convert methionine to cysteine, enabling them to escape ferroptosis [41]. In contrast, when other amino acids are deficient (at levels still sufficient to stimulate cell proliferation), cystine deprivation can effectively induce ferroptosis for anticancer therapy [42]. Cysteine can be catabolized into acetyl-CoA and taurine through two main pathways. Interestingly, acetyl-CoA can synergize with GSH to exert an anti-ferroptosis effect [43], while the production of taurine reduces intracellular GSH levels and increases ROS production [44]. This may be related to the fact that CoQ10, a CoA derivative, is reduced to panthenol by the oxidoreductase FSP1, thus preventing lipid peroxidation, while taurine production competes with cysteine for GSH synthesis. Additionally, cystine/cysteine can regulate GPX4 protein synthesis by activating the Rag-mTORC1-4EBPs signaling axis in a GSH-independent manner, thereby preventing ferroptosis [45].

As the most abundant amino acid in the human body and an important fuel, glutamine metabolism is also closely linked to ferroptosis. Glutamine can be converted to glutamate by glutaminase, and glutamate can combine with cysteine and glycine to synthesize GSH [46]. Glutamine can act as an inducer of ferroptosis, promoting ferroptosis through its own hydrolysis, but this process requires cystine starvation [47]. As essential amino acids, elevated serum levels of branched-chain amino acids, including leucine, valine, and isoleucine, increase ROS production by activating the Akt-mTOR signaling pathway, thereby affecting ferroptosis [48]. Tryptophan metabolites, such as 5-hydroxytryptamine and 3- hydroxyanthranilic acid, act as potential radical-trapping antioxidants and can protect cells from ferroptosis by eliminating lipid peroxidation [49]. Moreover, interleukin-4-induced-1, an amino acid oxidase secreted by immune cells, can inhibit ferroptosis through indole-3-pyruvate produced from tryptophan metabolism [26]. L-arginine can alleviate the oxidative stress induced by lipopolysaccharide (LPS). L-arginine increases the cellular GPX4 content and decreases ROS production through the arginase-1 signaling pathway, suggesting its involvement in the regulation of ferroptosis [50]. Furthermore, a study has demonstrated that lysine oxidase can activate ferroptosis signals through the oxidative deamination of lysine and the rapid generation of ROS [51].

## Gut microbiota and host metabolic homeostasis

### Host lipid metabolism

The gut microbiota plays an important role in host lipid metabolism process. Their crosstalk is essential for the development of various diseases, including obesity, alcoholic liver disease and other metabolic diseases [52, 53]. The gut microbiota can influence the composition of lipids in the host’s serum, adipose tissue, and liver, particularly triglycerides and phosphatidylcholine, thereby impacting the host’s energy and lipid metabolism [54]. For instance, *Lactobacillus rhamnosus* can regulate the abundance of beneficial bacteria in the gut microbiota of zebrafish larvae, thereby affecting the transcription of genes related to cholesterol-triglyceride metabolism and modulating host lipid processing and metabolism [55].

Researchers have focused on elucidating the specific mechanisms by which the gut microbiota affects host lipid metabolism. A previous study demonstrated that the circadian transcription factor NFIL3, which controls the expression of circadian-clock genes, may be a crucial molecule in the influence of the gut microbiota on host lipid metabolism and body composition [56]. The addition of exogenous melatonin improves the intestinal microbial composition and circadian rhythm in high-fat diet-fed mice, thereby regulating the host’s metabolic circadian clock and lipid metabolism [57]. Moreover, metabolites derived from the gut microbiota, such as SCFAs, secondary bile acids, triethylamine, and LPS, may mediate the regulation of host lipid metabolism and possess anti-obesity potential [58, 59]. In particular, free fatty acids and SCFAs play significant roles in the microbiota-host lipid metabolism axis, which has important implications for human health [60, 61]. In addition, specific strains of the gut microbiota can impact host lipid metabolism differently and have individual effects on disease progression [62]. For instance, *Lactobacillus paracasei* promotes lipid storage in intestinal cells, while *Escherichia coli* enhances lipid catabolism and ultimately inhibits lipid secretion [63]. *Escherichia fergusonii* interferes with host lipid metabolism by secreting microRNA-sized small RNAs that inhibit hepatic lipid β-oxidation and promote liver fat accumulation [64]. Further research in this field may provide valuable insights into potential therapeutic interventions for lipid-related diseases.

### Host iron homeostasis

The gut microbiota is involved in the absorption of iron in the host intestine, and microbial metabolites are associated with the regulation of iron homeostasis. Specifically, the function of the gut microbiota impacts host iron absorption, while iron homeostasis in the host also influences the diversity, classification, function, and dominant bacterial community of the microbiota [65]. A study in diabetic mice showed that iron metabolism is closely related to the abundance of *Lactobacillus* in the gut microbiota [66]. A comparative study of germ-free and microbially colonized mice indicated that the gut microbiota can induce specific iron-related protein signaling pathways and alter the perception of iron by intestinal epithelial cells [67]. Commensal bacteria secrete the siderophore enterobactin, which binds to the α subunit of ATP synthase, facilitating host mitochondria iron uptake [68]. This potential host mechanism of beneficial enterobactin utilization contributes to the maintenance of iron homeostasis in the host. Interestingly, gut microbiota-derived metabolites can reduce iron absorption in the host intestine by suppressing intestinal hypoxia-inducible factor-2α activity and decreasing ferritin expression [69]. Moreover, in a whole-body iron overload mouse model, hypoxia-inducible factor-2α inhibitory microbial metabolites efficiently prevented tissue iron accumulation.

Iron depletion in the gut lumen can alter the function of intestinal epithelial cells and the composition of the intestinal microbiota [70], while different iron supplements also lead to differences in the regulation of the gut microbiota [71]. Compared with those in iron-sufficient rats, cecal butyrate and propionate levels are significantly lower in iron-deficient rats, and the abundance of dominant species is noticeably altered [72]. Furthermore, iron sulfate supplementation had a stronger effect on the gut microbiota than electrolytic iron, indicating that ferrous iron was more readily utilized by the microbiota. Interestingly, under iron-rich conditions and in the absence of host influences, iron has adverse effects, including a decrease in beneficial microorganisms and an increase in bacterial metabolite levels, which in turn impairs the barrier function of intestinal epithelial cells [73]. Different doses and regimens of iron supplements have been suggested to have opposite effects. For example, ferrous bisglycinate is beneficial in sodium sulfate-induced colitis, whereas ferric ethylenediaminetetraacetic acid is highly detrimental [74]. Additionally, oral supplementation with ferrous sulfate can enhance the beneficial effects of probiotics on colitis. In general, understanding the intricate interactions between the gut microbiota and host iron metabolism can provide valuable insights into interventions for iron-related disorders and the maintenance of intestinal health.

### Host amino acid metabolism

The gut microbiota performs several functions in the host’s life, including the conversion of nutrients that the host cannot digest into usable compounds. It can provide the host with essential amino acids necessary for tissue structure synthesis and may also play a significant role in amino acid metabolism in protein-deficient animals [75]. The alteration of amino acid metabolism in the host brain was presented based on the differentiated amino acid concentrations of plasma and brain between conventionally raised mice and germ-free mice, which attributed to the participation of gut microbiota [76]. Moreover, studies based on gene expression data and tissue-specific genome-scale metabolic models have demonstrated that the gut microbiota can influence host amino acid metabolism and contribute to changes in glutathione metabolism [77]. Similarly, experiments with different concentrations of antibiotics for bumblebees have shown that the gut microbiota may regulate host growth through essential amino acids and BCAAs [78]. For instance, supplementation with BCAAs can alleviate gut microbial dysbiosis, improving amino acid metabolism and host growth performance [79]. The impact of the gut microbiota on host amino acid metabolism can further influence disease progression and therapeutic response [80]. Understanding the complicated relationship between the gut microbiota and amino acid metabolism is critical for elucidating disease progression and optimizing therapeutic interventions.

## Advanced studies on the correlation between the gut microbiota and ferroptosis

**Fig. 2:**
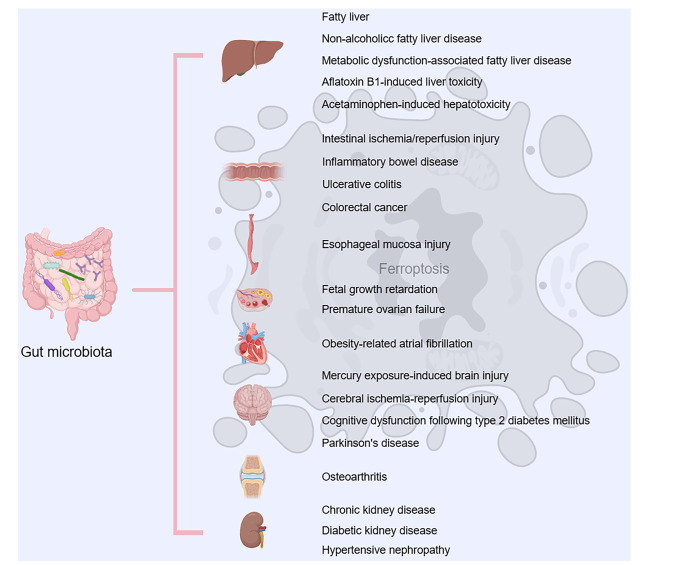
The gut microbiota has been implicated on ferroptosis in a variety of organs, tissues, and diseases. The gut microbiota is associated with ferroptosis in various tissues and organs, such as the liver, intestines, and kidneys, contributing to disease progression.

In recent years, there has been a growing focus on the role of the gut microbiota in the occurrence of ferroptosis, with the aim of elucidating the potential relationship between the microbiota and ferroptosis (Fig. 2). For instance, a recent study revealed that microbial metabolic disorders leading to Th17/Treg imbalance can trigger hepatocyte ferroptosis, potentially explaining the involvement of *Porphyromonas gingivalis* in the progression of non-alcoholic fatty liver disease [81]. Obeticholic acid, a clinical drug for non-alcoholic fatty liver disease treatment, can induce liver lipid peroxidation by affecting the gut microbiota, which subsequently mediates hepatocyte ferroptosis and compromises its antioxidative and antifibrotic effects [82]. In the presence of environmental toxins, gut microbiota-derived metabolites (such as glycochenodeoxycholate) can induce ferroptosis, lipid metabolic disorders, and inflammation by activating the TFR and ACSL4 [83]. Exogenous toxins also increase the synthesis of LPS, intensifying hepatocyte ferroptosis and resulting in hepatic lipid metabolic disorders. However, melatonin can decrease microbiota-derived LPS and hepatocyte ferroptosis by modulating the gut microbiota [84]. The total flavonoids of Rhizoma Drynaria can prevent oxidative stress and hepatic lipid deposition, reverse hepatic ferroptosis by improving the host intestinal microenvironment, and play a protective role in aflatoxin B1-induced liver damage [85]. Daidzein, released by gut microbial β-galactosidases, can suppress hepatocyte ferroptosis by reducing farnesyl diphosphate synthase expression [86]. Urolithin C can alleviate the adverse effects of a choline-deficient amino acid-defined high-fat diet by regulating the AMPK-ferroptosis axis, which involves the gut-liver axis. Furthermore, the application of urolithin C can ameliorate intestinal mucosal barrier and gut microbiota dysbiosis [87]. These studies highlight therapeutic interventions targeting the gut microbiota, which are expected to alleviate hepatic lipid metabolism disorders and ferroptosis-associated liver damage.

Ferroptosis and the gut microbiota are closely related in the context of gastrointestinal disease. A previous study indicated that capsiate enhances GPX4 expression and suppresses ferroptosis through the activation of transient receptor potential cation channel subfamily V member 1, along with the attenuation of intestinal I/R injury [88]. Ferroptosis caused by microbiota dysbiosis can be inhibited by early-life gut microbiota-derived ether lipids (plasmalogens), which also influence susceptibility to colitis [89]. The effect of maternal embryonic leucine zipper kinase pharmacological inhibitors, which suppress ferroptosis in intestinal tissue and alleviate intestinal inflammation in mice with colitis, is closely related to the regulation of the gut microbiota [90]. The therapeutic effect of protocatechuic acid on ulcerative colitis is accomplished by regulating the gut microbiota and suppressing ferroptosis [91]. In addition to inhibiting ferroptosis, the iron chelator deferasirox also improves gut microbiota dysbiosis and enhances the production of SCFAs during dextran sulfate sodium salt-induced ulcerative colitis [92]. Similarly, ferrostatin-1 can restore gut microbiota homeostasis and alleviate ionizing radiation-induced intestinal injury by inhibiting ferroptosis and the p53-mediated apoptosis signaling pathway [93]. A recent study illustrated that trans-3-indoleacrylic acid, a tryptophan metabolite derived from the intestinal microbe *P. anaerobius*, accelerates colorectal carcinogenesis by inhibiting ferroptosis [94]. These findings highlight the complex role of understanding the regulation of the gut microbiota in the development and treatment of gastrointestinal diseases related to ferroptosis, contributing to the elucidation of potential mechanisms underlying gastrointestinal diseases and the exploration of new therapeutic approaches.

The gut microbiota has a significant impact on ferroptosis and stability in other parts of the host, beyond just the liver and intestine. Gut microbiota dysbiosis and microbiota-derived metabolites interfere with the regulation of ferroptosis. For example, dysbiosis of the esophageal/intestinal microbiome and elevation of peripheral blood LPS can regulate ferroptosis in the esophageal epithelium by increasing ACSL4 expression, serum ferritin secretion, and iron accumulation [95]. Gut microbiota dysbiosis caused by perfluorooctanoic acid can lead to fetal growth retardation through ferroptosis and inflammation [96]. Obesity-related atrial fibrillation susceptibility is increased by gut microbiota dysbiosis, which activates the toll-like receptor 4/nuclear factor kappa-B/NOD-like receptor thermal protein domain associated protein 3 signaling pathway and induces ferroptosis [97]. Mercury exposure-induced brain injury in common carp is associated with neuronal ferroptosis via the augmentation of intestinal *A.hydrophila* [98]. Additionally, the gut microbiota-derived metabolite capsiate relieves ferroptosis-related osteoarthritis through the regulation of solute carrier family 2 member 1 and HIF-1α [99]. On the other hand, interventions targeting the gut microbiota, such as berberine supplementation can suppress neuronal ferroptosis by modulating the gut microbiota and mitigating cerebral I/R injury [100]. Similarly, the neuroprotective effect of sinomenine on cognitive dysfunction following type 2 diabetes mellitus is achieved through the inhibition of ferroptosis in hippocampal neurons via the classical ferroptosis signaling pathway (epidermal growth factor/nuclear factor erythroid derived 2-related factor 2 (Nrf2)/heme oxygenase 1) and the microbiota-gut-brain axis [101]. Furthermore, electroacupuncture inhibits ovarian oxidative stress and Fe2^+^ accumulation in premature ovarian failure mice by altering the gut microbiota [102]. Probiotic supplementation can reverse bacteroidaceae-induced intestinal ferroptosis, thereby relieving systemic inflammation and hematopoietic toxicity [7]. Moreover, the use of probiotics inhibits perfluorobutanesulfonate-mediated ferroptosis and improves metabolic disorders in the host [103]. The next-generation probiotic strain *L. lactis* MG1363-pMG36e-GLP-1 plays a neurotrophic role in Parkinson’s disease through the regulation of oxidative stress, prevention of ferroptosis, and correction of dysbiosis [104]. In conclusion, the complex relationship between the gut microbiota and ferroptosis emphasizes the importance of maintaining a healthy microbiota for host health. Further research in this field holds the potential to develop new therapeutic strategies for iron-related diseases.

## Potential mechanisms of gut microbiota interaction with ferroptosis

### System Xc^-^/GPX4 axis

System Xc^-^, also known as the cystine-glutamate reverse transporter, is primarily responsible for the intracellular and extracellular transport of amino acids [105]. Specifically, it transports intracellular glutamate out of the cell and takes up extracellular cystine, which is then reduced to cysteine. It consists of the transporter proteins solute carrier family 7 member 11 and solute carrier family 3 member 2. System Xc^-^ plays a critical role in maintaining the body’s levels of GSH by participating in glutamate release, cystine uptake, and GSH synthesis. GSH is an important antioxidant in mammalian cells and is synthesized from glutamate, cysteine, and glycine [106]. GSH functions as a prosthetic group of glyceraldehyde phosphate dehydrogenase and as a coenzyme of glyoxalase and triose dehydrogenase. It is involved in the tricarboxylic acid cycle, glucose metabolism, and can activate various enzymes to promote glycometabolism, lipid metabolism, and protein metabolism. Additionally, the active sulfhydryl group carried by GSH can combine with free radicals in the body, eliminate oxidants, and participate in redox reactions. GPX4, a crucial oxidoreductase, is essential for removing lipid peroxidation products by using GSH as a reducing agent [107]. GPX4 converts GSH to oxidized glutathione, resulting in a decrease in the peroxidation of PUFAs. Undoubtedly, GPX4 is a key suppressor of ferroptosis. Inactivation of system Xc^-^ or dysfunction in GSH synthesis leads to reduced GPX4 activity, impairing cellular antioxidant capacity and ultimately resulting in ferroptosis.

A study on the intestinal microbiome of young and old mice has revealed that the gut microbial-derived metabolite 3-hydroxyphenylacetic acid upregulates GPX4 expression, inhibits ferroptosis, and alleviates spermatogenic dysfunction in aging mice [108]. Another gut microbial-derived metabolite, capsiate, alleviates ferroptosis induced by intestinal I/R injury by promoting GPX4 expression through the activation of transient receptor potential cation channel subfamily V member 1 [88]. However, dysbiosis of the gut microbiota, leading to the colonization of harmful bacteria such as *adherent-invasive E. coli*, exacerbates lipid peroxidation and ferroptosis in intestinal epithelial cells by reducing GPX4 and ferritin heavy chain levels [109]. In a study on the alleviation of kidney injury using the traditional Chinese medicine Mori Fructus aqueous extracts, it was found that extracts altered the gut microbiota in experimental mice, accelerated the nuclear transport of Nrf2, and increased the expression of heme oxygenase 1 and GPX4. Nonetheless, there is a lack of direct evidence linking the increase in Nrf2 to alterations in the gut microbiota. Another recent study confirmed that gut microbiota-derived butyrate improves ferroptosis in mice with ulcerative colitis through the Nrf2/GPX4 signaling pathway and protects the integrity of the intestinal mucosal barrier [110]. Fecal microbiota transplantation experiments further verified that the regulation of the gut microbiota can upregulate the Nrf2/GPX4 pathway to attenuate ferroptosis in septic liver injury [111].

### FSP1/CoQ10/NADPH system

FSP1, identified as another ferroptosis suppressor through genome-wide screening, primarily prevents lipid peroxidation and ferroptosis by reducing lipid free radicals in lipid droplets or plasma membranes [112]. The FSP1/CoQ10/NADPH system protects cells from ferroptosis in a manner independent of the GPX4 axis by inhibiting lipid peroxidation and ferroptosis through the NAD(P)H-dependent reduction of CoQ10 to ubiquinol (CoQH_2_) [23]. Recent research has demonstrated that trans-3-indoleacrylic acid, a tryptophan metabolite derived from the gut microbiota, suppresses ferroptosis and facilitates colorectal cancer progression [94]. Metabolite-dependent resistance to ferroptosis relies on the FSP1/CoQ10/NADPH system. Specifically, as an endogenous ligand of the aromatic hydrocarbon receptor, trans-3-indoleacrylic acid upregulates the expression of aldehyde dehydrogenase 1 family member A3, leading to increased production of NADH by utilizing retinol as a substrate. Furthermore, it promotes FSP1-mediated synthesis of reductive CoQ10 and inhibits ferroptosis in tumor cells. During disease progression, alterations in the gut microbiota are accompanied by functional changes in GSH metabolism and CoQ10 biosynthesis pathways [113]. Moreover, some quinones, particularly menaquinones, are essential growth factors for the gut microbiota and regulate microbiota homeostasis by improving the growth medium and promoting symbiotic bacterial growth [114]. CoQ10-rich pumpkin juice obtained through the fermentation of *Rhodobacter sphaeroides* not only exhibits antioxidant capacity, especially ferric ion reduction antioxidant capacity but also modulates the gut microbiota of mammals to protect the intestinal barrier [115]. These studies highlight the crucial role of CoQ10 in lipid peroxidation, ferroptosis, and microbiota growth.

### GCH1/BH4/dihydrofolate reductase (DHFR) system

The GCH1/BH4/DHFR system is a novel mechanism for suppressing ferroptosis that operates independently of GPX4 [25]. BH4 promotes the synthesis of CoQ10 by converting phenylalanine to tyrosine, thereby serving as a free radical-trapping antioxidant [116]. Additionally, GCH1, a rate-limiting enzyme synthesized by BH4, has the potential to suppress ferroptosis [117]. The expression of GCH1 induces lipid remodeling in cells, inhibiting ferroptosis by selectively preventing the consumption of PLs with two PUFA tails [25]. DHFR, a component of the BH4 recycling process, can cooperate with GPX4 inhibitors to promote the occurrence of ferroptosis. A study suggested that in GCH1-deficient mice, BH4 could still accumulate with age, which was associated with the production of BH4 by specific gut microbiota, such as intestinal *Actinobacteria* [118]. BH4 derived from the microbiota also stimulates the gut microbiota to enhance the production of L-DOPA, thereby improving brain function and behavior [119, 120]. Therefore, interventions targeting the gut microbiota may increase the levels of BH4 in the body, which can be beneficial for human health, especially for patients with congenital biopterin deficiency. Furthermore, the diversity of gut microbiota taxa and the abundance of bacterial gene families are associated with the inhibitory effect of drugs on DHFR [121].

### DHODH/CoQH_2_ system

DHODH is located on the inner mitochondrial membrane and its primary function is to catalyze the synthesis of pyrimidine nucleotides. During this process, DHODH oxidizes dihydroorotate to orotate, while CoQ10 receives electrons and is reduced to CoQH_2_ [24]. CoQH_2_ is a free radical-trapping antioxidant that prevents lipid peroxidation and inhibits ferroptosis. Similar to FSP1, DHODH is a CoQ10-reduced flavin protein that acts as a GPX4-independent defense system against ferroptosis in mitochondria. Moreover, GPX4 downregulation limits mitochondrial lipid peroxidation and ferroptosis [122]. The use of metformin in patients with gestational diabetes mellitus affects the composition and metabolic characteristics of the gut microbiota, particularly the metabolic pathways related to CoQH_2_ biosynthesis [123]. However, further studies are needed to establish a direct relationship between CoQH_2_ synthesis and the therapeutic effects of metformin. In a study on aging-related diseases, an increase in the abundance of gut microbiome genes involved in the CoQH_2_ synthesis pathway was observed with age [124]. Nevertheless, a more in-depth discussion of the underlying mechanisms is still lacking.

## Using CKD as an example: bibliometric analysis of ferroptosis and the gut microbiota

We conducted a literature search on the Web of Science Core Collection (https://www.webofscience.com/wos/woscc/basic-search) from January 1, 2012 (the year when the concept of ferroptosis was introduced), to January 28, 2024. The search criteria were as follows: 1st: (TS = (ferroptosis)) AND TS = (chronic kidney disease); 2nd: (TS = (gut microbiota)) AND TS = (chronic kidney disease); 3rd: (TS = (ferroptosis)) AND TS = (gut microbiota). We selected articles of the “Article” and “Review Article” types and limited the search to articles published in English. This resulted in a total of 1113 articles on ferroptosis and the gut microbiota in the context of CKD. Subsequently, we utilized Microsoft Office Excel 2019, CiteSpace, VOSviewer, and the R package “bibliometrix” for analysis and visualization.

**Fig. 3:**
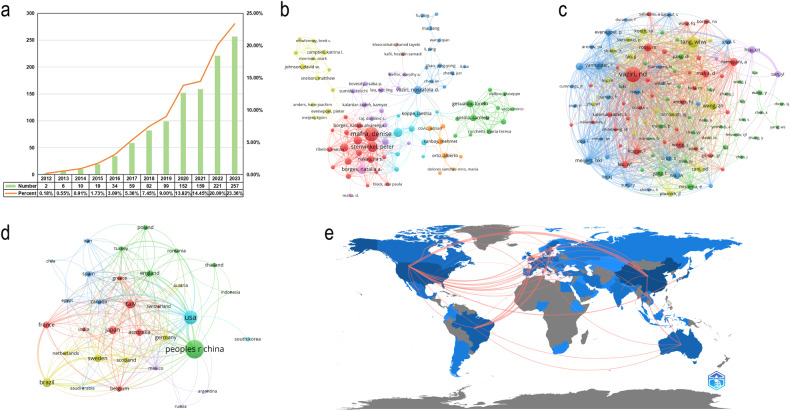
Analysis of publication quantity, country, authors, and co-cited authors. **a** annual quantity of publications; **b** Visualization of the co-authorship author network; **c** Visualization of the co-citation cited author network; **d** Visualization of the co-authorship country network; **e** country collaboration map.

**Fig. 4:**
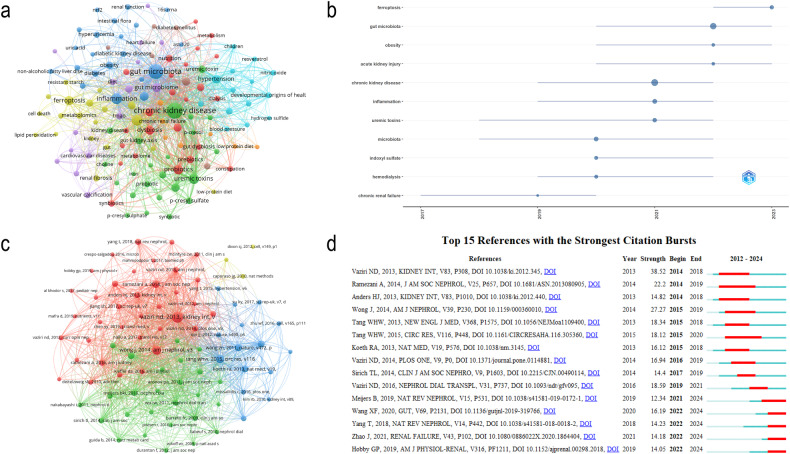
Analysis of keywords and references. **a** Visualization of the co-occurrence author’s keyword network; **b** trend topic analysis; **c** Visualization of the co-citation cited reference network; **d** Top 15 references with the strongest citation bursts. A red bar indicates a high number of citations in that year.

Figure 3a displays the increasing number of publications on ferroptosis and the gut microbiota in CKD over the past decade, with a particularly notable increase in the last 5 years. Numerous scholars have dedicated their efforts to this research area, and close collaboration among them is evident (Fig. 3b). Similarly, a positive and strong cooperative relationship exists between co-cited authors (Fig. 3c). The majority of these publications originate from Asia, North America, and Europe, including countries such as China, the United States, and Italy. Notably, there is substantial collaboration among different countries (Fig. 3d, e). Co-occurrence analysis of authors’ keywords indicates that metabolism, dysbiosis, inflammation, and uremic toxins are the primary research directions in the field of ferroptosis and gut microbiota in CKD (Fig. 4a). Keyword trend topic analysis demonstrates that ferroptosis and the gut microbiota have emerged as focal points in CKD research in recent years (Fig. 4b). Finally, the co-cited references network and references with citation bursts provide insight into the frequently cited literature in this field and the co-citation relationships among them (Fig. 4c, d). The majority of the top 15 cited references focus on the gut microbiota and CKD. This could be attributed to the emergence of the concept of ferroptosis only within the last decade, with ongoing research exploring its role in CKD each year. The top 15 references with strongest citation bursts show alterations in the gut microbiota in CKD patients and animal models [125, 126], characterized by a decrease in bacteria-producing SCFAs and an increase in bacteria-producing uremic toxins [127]. Dysbiosis of the gut microbiota disrupts the structure and function of the intestinal epithelial barrier [128], forming harmful metabolic profiles [129], which induce inflammation and immune dysfunction via the gut-kidney axis [130, 131], exacerbating renal injury [132]. CKD patients should consume more foods rich in dietary fiber [133, 134] and avoid high-choline foods such as L-carnitine [135] and phosphatidylcholine [136] because trimethylamine-N-oxide, generated from choline metabolism by the gut microbiota, contributes to the progression of CKD and the risk of death [137]. In conclusion, targeting the gut microbiota is a novel therapeutic direction to improve CKD outcomes, and interventions such as probiotics to modulate the gut microbiota may provide new insights into individualized CKD treatment.

## Summary and perspective

Ferroptosis is a regulated, iron-dependent, lipid peroxidation-driven cell death process that has gained increasing attention in recent years. Alterations in the gut microbiota can impact host metabolic homeostasis and the antioxidant system, suggesting that the gut microbiota and its metabolites may regulate ferroptosis. However, despite of numerous studies indirectly demonstrating a link between ferroptosis and the gut microbiota, there is a lack of robust evidence directly supporting the causal relationship. Furthermore, the specific role of the gut microbiota in regulating ferroptosis and the key regulatory factors involved require further discussion. Additionally, we provide a summary of studies on ferroptosis and the gut microbiota in CKD, aiming to identify meaningful clues for investigating disease mechanisms and developing treatment strategies. Currently, there are several unresolved questions that warrant further study:

1. Role of specific microbiota and metabolites: Identifying which specific microbiota species and metabolites play a regulatory role in ferroptosis and determining whether their impact on ferroptosis is positive or negative.
2. Mechanisms of gut microbiota modulation: Investigating the influence of the gut microbiota on the defense system against ferroptosis and the signaling pathways through which the gut microbiota regulates ferroptosis.
3. Influence of interventions: Exploring the effects of interventions that regulate the gut microbiota, such as probiotics, prebiotics, and fecal microbiota transplantation, on ferroptosis, as well as the therapeutic implications of these interventions for ferroptosis-related diseases.

By addressing these problems, a deeper understanding of the influence of the gut microbiota on ferroptosis and its implications for various diseases can be gained, paving the way for potential therapeutic interventions and personalized treatment approaches.

## References

[1] Dixon SJ, Lemberg KM, Lamprecht MR, Skouta R, Zaitsev EM, Gleason CE (2012). Ferroptosis: an iron-dependent form of nonapoptotic cell death. Cell.

[2] Bayır H, Dixon SJ, Tyurina YY, Kellum JA, Kagan VE (2023). Ferroptotic mechanisms and therapeutic targeting of iron metabolism and lipid peroxidation in the kidney. Nat Rev Nephrol.

[3] Mao K, Baptista AP, Tamoutounour S, Zhuang L, Bouladoux N, Martins AJ (2018). Innate and adaptive lymphocytes sequentially shape the gut microbiota and lipid metabolism. Nature.

[4] Zhang J, Ankawi G, Sun J, Digvijay K, Yin Y, Rosner MH (2018). Gut-kidney crosstalk in septic acute kidney injury. Crit Care.

[5] Mao Z-H, Gao Z-X, Liu D-W, Liu Z-S, Wu P (2023). Gut microbiota and its metabolites—molecular mechanisms and management strategies in diabetic kidney disease. Front Immunol.

[6] Yao T, Li L (2023). The influence of microbiota on ferroptosis in intestinal diseases. Gut Microbes.

[7] Zhang L, Kang H, Zhang W, Wang J, Liu Z, Jing J (2023). Probiotics ameliorate benzene-induced systemic inflammation and hematopoietic toxicity by inhibiting Bacteroidaceae-mediated ferroptosis. Sci Total Environ.

[8] Gansevoort RT, Correa-Rotter R, Hemmelgarn BR, Jafar TH, Heerspink HJL, Mann JF (2013). Chronic kidney disease and cardiovascular risk: epidemiology, mechanisms, and prevention. Lancet.

[9] Mitrofanova A, Merscher S, Fornoni A (2023). Kidney lipid dysmetabolism and lipid droplet accumulation in chronic kidney disease. Nat Rev Nephrol.

[10] Krukowski H, Valkenburg S, Madella A-M, Garssen J, van Bergenhenegouwen J, Overbeek SA (2023). Gut microbiome studies in CKD: opportunities, pitfalls and therapeutic potential. Nat Rev Nephrol.

[11] Stockwell BR (2022). Ferroptosis turns 10: emerging mechanisms, physiological functions, and therapeutic applications. Cell.

[12] Pope LE, Dixon SJ (2023). Regulation of ferroptosis by lipid metabolism. Trends Cell Biol.

[13] Liang D, Minikes AM, Jiang X (2022). Ferroptosis at the intersection of lipid metabolism and cellular signaling. Mol Cell.

[14] Dixon SJ, Winter GE, Musavi LS, Lee ED, Snijder B, Rebsamen M (2015). Human haploid cell genetics reveals roles for lipid metabolism genes in nonapoptotic cell death. ACS Chem Biol.

[15] Doll S, Proneth B, Tyurina YY, Panzilius E, Kobayashi S, Ingold I (2017). ACSL4 dictates ferroptosis sensitivity by shaping cellular lipid composition. Nat Chem Biol.

[16] Zhang H-L, Hu B-X, Li Z-L, Du T, Shan J-L, Ye Z-P (2022). PKCβII phosphorylates ACSL4 to amplify lipid peroxidation to induce ferroptosis. Nat Cell Biol.

[17] Minami JK, Morrow D, Bayley NA, Fernandez EG, Salinas JJ, Tse C (2023). CDKN2A deletion remodels lipid metabolism to prime glioblastoma for ferroptosis. Cancer Cell.

[18] Chen D, Chu B, Yang X, Liu Z, Jin Y, Kon N (2021). iPLA2β-mediated lipid detoxification controls p53-driven ferroptosis independent of GPX4. Nat Commun.

[19] Li D, Li Y (2020). The interaction between ferroptosis and lipid metabolism in cancer. Signal Transduct Target Ther.

[20] Cao JY, Poddar A, Magtanong L, Lumb JH, Mileur TR, Reid MA (2019). A genome-wide haploid genetic screen identifies regulators of glutathione abundance and ferroptosis sensitivity. Cell Rep.

[21] Kang YP, Mockabee-Macias A, Jiang C, Falzone A, Prieto-Farigua N, Stone E (2021). Non-canonical glutamate-cysteine ligase activity protects against ferroptosis. Cell Metab.

[22] Shimada K, Skouta R, Kaplan A, Yang WS, Hayano M, Dixon SJ (2016). Global survey of cell death mechanisms reveals metabolic regulation of ferroptosis. Nat Chem Biol.

[23] Bersuker K, Hendricks JM, Li Z, Magtanong L, Ford B, Tang PH (2019). The CoQ oxidoreductase FSP1 acts parallel to GPX4 to inhibit ferroptosis. Nature.

[24] Mao C, Liu X, Zhang Y, Lei G, Yan Y, Lee H (2021). DHODH-mediated ferroptosis defence is a targetable vulnerability in cancer. Nature.

[25] Kraft VAN, Bezjian CT, Pfeiffer S, Ringelstetter L, Müller C, Zandkarimi F (2020). GTP cyclohydrolase 1/tetrahydrobiopterin counteract ferroptosis through lipid remodeling. ACS Cent Sci.

[26] Zeitler L, Fiore A, Meyer C, Russier M, Zanella G, Suppmann S (2021). Anti-ferroptotic mechanism of IL4i1-mediated amino acid metabolism. Elife.

[27] Viswanathan VS, Ryan MJ, Dhruv HD, Gill S, Eichhoff OM, Seashore-Ludlow B (2017). Dependency of a therapy-resistant state of cancer cells on a lipid peroxidase pathway. Nature.

[28] Shah R, Shchepinov MS, Pratt DA (2018). Resolving the role of lipoxygenases in the initiation and execution of ferroptosis. ACS Cent. Sci.

[29] Gao M, Monian P, Pan Q, Zhang W, Xiang J, Jiang X (2016). Ferroptosis is an autophagic cell death process. Cell Res.

[30] Chen P-H, Wu J, Ding C-KC, Lin C-C, Pan S, Bossa N (2020). Kinome screen of ferroptosis reveals a novel role of ATM in regulating iron metabolism. Cell Death Differ.

[31] Yu F, Zhang Q, Liu H, Liu J, Yang S, Luo X (2022). Dynamic O-GlcNAcylation coordinates ferritinophagy and mitophagy to activate ferroptosis. Cell Discov.

[32] Liu J, Song X, Kuang F, Zhang Q, Xie Y, Kang R (2021). NUPR1 is a critical repressor of ferroptosis. Nat Commun.

[33] Wang H, An P, Xie E, Wu Q, Fang X, Gao H (2017). Characterization of ferroptosis in murine models of hemochromatosis. Hepatology.

[34] Wu Y, Jiao H, Yue Y, He K, Jin Y, Zhang J (2022). Ubiquitin ligase E3 HUWE1/MULE targets transferrin receptor for degradation and suppresses ferroptosis in acute liver injury. Cell Death Differ.

[35] Fuhrmann DC, Mondorf A, Beifuß J, Jung M, Brüne B (2020). Hypoxia inhibits ferritinophagy, increases mitochondrial ferritin, and protects from ferroptosis. Redox Biol.

[36] Li K, Chen B, Xu A, Shen J, Li K, Hao K (2022). TRIM7 modulates NCOA4-mediated ferritinophagy and ferroptosis in glioblastoma cells. Redox Biol.

[37] Yao F, Peng J, Zhang E, Ji D, Gao Z, Tang Y (2023). Pathologically high intraocular pressure disturbs normal iron homeostasis and leads to retinal ganglion cell ferroptosis in glaucoma. Cell Death Differ.

[38] Oliveira T, Hermann E, Lin D, Chowanadisai W, Hull E, Montgomery M (2021). HDAC inhibition induces EMT and alterations in cellular iron homeostasis to augment ferroptosis sensitivity in SW13 cells. Redox Biol.

[39] Jiang X, Stockwell BR, Conrad M (2021). Ferroptosis: mechanisms, biology and role in disease. Nat Rev Mol Cell Biol.

[40] Dixon SJ, Patel DN, Welsch M, Skouta R, Lee ED, Hayano M (2014). Pharmacological inhibition of cystine-glutamate exchange induces endoplasmic reticulum stress and ferroptosis. Elife.

[41] Floros KV, Chawla AT, Johnson-Berro MO, Khatri R, Stamatouli AM, Boikos SA (2022). MYCN upregulates the transsulfuration pathway to suppress the ferroptotic vulnerability in MYCN-amplified neuroblastoma. Cell Stress.

[42] Conlon M, Poltorack CD, Forcina GC, Armenta DA, Mallais M, Perez MA (2021). A compendium of kinetic modulatory profiles identifies ferroptosis regulators. Nat Chem Biol.

[43] Badgley MA, Kremer DM, Maurer HC, DelGiorno KE, Lee H-J, Purohit V (2020). Cysteine depletion induces pancreatic tumor ferroptosis in mice. Science.

[44] Kang YP, Torrente L, Falzone A, Elkins CM, Liu M, Asara JM (2019). Cysteine dioxygenase 1 is a metabolic liability for non-small cell lung cancer. Elife.

[45] Zhang Y, Swanda RV, Nie L, Liu X, Wang C, Lee H (2021). mTORC1 couples cyst(e)ine availability with GPX4 protein synthesis and ferroptosis regulation. Nat Commun.

[46] Yang J, Dai X, Xu H, Tang Q, Bi F (2022). Regulation of ferroptosis by amino acid metabolism in cancer. Int J Biol Sci.

[47] Gao M, Monian P, Quadri N, Ramasamy R, Jiang X (2015). Glutaminolysis and transferrin regulate ferroptosis. Mol Cell.

[48] Zhenyukh O, Civantos E, Ruiz-Ortega M, Sánchez MS, Vázquez C, Peiró C (2017). High concentration of branched-chain amino acids promotes oxidative stress, inflammation and migration of human peripheral blood mononuclear cells via mTORC1 activation. Free Radic Biol Med.

[49] Liu D, Liang C-H, Huang B, Zhuang X, Cui W, Yang L (2023). Tryptophan metabolism acts as a new anti-ferroptotic pathway to mediate tumor growth. Adv. Sci.

[50] Qiu Y, Yang X, Wang L, Gao K, Jiang Z (2019). L-arginine inhibited inflammatory response and oxidative stress induced by lipopolysaccharide via arginase-1 signaling in IPEC-J2 cells. Int J. Mol. Sci.

[51] Chepikova OE, Malin D, Strekalova E, Lukasheva EV, Zamyatnin AA, Cryns VL (2020). Lysine oxidase exposes a dependency on the thioredoxin antioxidant pathway in triple-negative breast cancer cells. Breast Cancer Res Treat.

[52] Su H, Yuan P, Lei H, Zhang L, Deng D, Zhang L (2022). Long-term chronic exposure to di-(2-ethylhexyl)-phthalate induces obesity via disruption of host lipid metabolism and gut microbiota in mice. Chemosphere.

[53] Fang C, Zhou Q, Liu Q, Jia W, Xu Y (2022). Crosstalk between gut microbiota and host lipid metabolism in a mouse model of alcoholic liver injury by chronic baijiu or ethanol feeding. Food Funct.

[54] Velagapudi VR, Hezaveh R, Reigstad CS, Gopalacharyulu P, Yetukuri L, Islam S (2010). The gut microbiota modulates host energy and lipid metabolism in mice. J. Lipid Res.

[55] Falcinelli S, Picchietti S, Rodiles A, Cossignani L, Merrifield DL, Taddei AR (2015). Lactobacillus rhamnosus lowers zebrafish lipid content by changing gut microbiota and host transcription of genes involved in lipid metabolism. Sci Rep.

[56] Wang Y, Kuang Z, Yu X, Ruhn KA, Kubo M, Hooper LV (2017). The intestinal microbiota regulates body composition through NFIL3 and the circadian clock. Science.

[57] Yin J, Li Y, Han H, Ma J, Liu G, Wu X (2020). Administration of exogenous melatonin improves the diurnal rhythms of the gut microbiota in mice fed a high-fat diet. mSystems.

[58] Schoeler M, Caesar R (2019). Dietary lipids, gut microbiota and lipid metabolism. Rev Endocr Metab Disord.

[59] Guo W, Zhu S, Li S, Feng Y, Wu H, Zeng M (2021). Microalgae polysaccharides ameliorates obesity in association with modulation of lipid metabolism and gut microbiota in high-fat-diet fed C57BL/6 mice. Int J Biol Macromol.

[60] Rodríguez-Carrio J, Salazar N, Margolles A, González S, Gueimonde M, de Los Reyes-Gavilán CG (2017). Free fatty acids profiles are related to gut microbiota signatures and short-chain fatty acids. Front Immunol.

[61] Lukovac S, Belzer C, Pellis L, Keijser BJ, de Vos WM, Montijn RC (2014). Differential modulation by Akkermansia muciniphila and Faecalibacterium prausnitzii of host peripheral lipid metabolism and histone acetylation in mouse gut organoids. mBio.

[62] Wu Q, Zhuang M, Guo T, Bao S, Wu S, Ke S (2023). Gut microbiota, host lipid metabolism and regulation mechanism of high-fat diet induced mice following different probiotics-fermented wheat bran intervention. Food Res Int.

[63] Tazi A, Araujo JR, Mulet C, Arena ET, Nigro G, Pédron T (2018). Disentangling host-microbiota regulation of lipid secretion by enterocytes: insights from commensals Lactobacillus paracasei and Escherichia coli. mBio.

[64] Xin F-Z, Zhao Z-H, Liu X-L, Pan Q, Wang Z-X, Zeng L (2022). Escherichia fergusonii promotes nonobese nonalcoholic fatty liver disease by interfering with host hepatic lipid metabolism through its own msRNA 23487. Cell Mol Gastroenterol Hepatol.

[65] Mayneris-Perxachs J, Moreno-Navarrete JM, Fernández-Real JM (2022). The role of iron in host-microbiota crosstalk and its effects on systemic glucose metabolism. Nat Rev Endocrinol.

[66] Shi J, Zhao Q, Hao DD, Miao HX, Wan S, Zhou CH (2022). Gut microbiota profiling revealed the regulating effects of salidroside on iron metabolism in diabetic mice. Front Endocrinol.

[67] Deschemin J-C, Noordine M-L, Remot A, Willemetz A, Afif C, Canonne-Hergaux F (2016). The microbiota shifts the iron sensing of intestinal cells. FASEB J.

[68] Qi B, Han M (2018). Microbial siderophore enterobactin promotes mitochondrial iron uptake and development of the host via interaction with ATP synthase. Cell.

[69] Das NK, Schwartz AJ, Barthel G, Inohara N, Liu Q, Sankar A (2020). Microbial metabolite signaling is required for systemic iron homeostasis. Cell Metab.

[70] Werner T, Wagner SJ, Martínez I, Walter J, Chang J-S, Clavel T (2011). Depletion of luminal iron alters the gut microbiota and prevents Crohn’s disease-like ileitis. Gut.

[71] Seyoum Y, Baye K, Humblot C (2021). Iron homeostasis in host and gut bacteria—a complex interrelationship. Gut Microbes.

[72] Dostal A, Chassard C, Hilty FM, Zimmermann MB, Jaeggi T, Rossi S (2012). Iron depletion and repletion with ferrous sulfate or electrolytic iron modifies the composition and metabolic activity of the gut microbiota in rats. J Nutr.

[73] Kortman GAM, Dutilh BE, Maathuis AJH, Engelke UF, Boekhorst J, Keegan KP (2015). Microbial metabolism shifts towards an adverse profile with supplementary iron in the TIM-2 in vitro model of the human colon. Front Microbiol.

[74] Constante M, Fragoso G, Lupien-Meilleur J, Calvé A, Santos MM (2017). Iron supplements modulate colon microbiota composition and potentiate the protective effects of probiotics in dextran sodium sulfate-induced colitis. Inflamm Bowel Dis.

[75] Newsome SD, Feeser KL, Bradley CJ, Wolf C, Takacs-Vesbach C, Fogel ML (2020). Isotopic and genetic methods reveal the role of the gut microbiome in mammalian host essential amino acid metabolism. Proc Biol Sci.

[76] Kawase T, Nagasawa M, Ikeda H, Yasuo S, Koga Y, Furuse M (2017). Gut microbiota of mice putatively modifies amino acid metabolism in the host brain. Br J Nutr.

[77] Mardinoglu A, Shoaie S, Bergentall M, Ghaffari P, Zhang C, Larsson E (2015). The gut microbiota modulates host amino acid and glutathione metabolism in mice. Mol Syst Biol.

[78] Chen R, Li L, Zhao W (2023). Antibiotics-induced dysbiosis in gut microbiota affects bumblebee health via regulating host amino acid metabolism. Amino Acids.

[79] Yin J, Ma J, Li Y, Ma X, Chen J, Zhang H (2020). Branched-chain amino acids, especially of leucine and valine, mediate the protein restricted response in a piglet model. Food Funct.

[80] Yang Q, Wei Y, Zhu Y, Guo J, Zhang J, He Y (2023). The interaction between gut microbiota and host amino acids metabolism in multiple myeloma. Cancers.

[81] Yao C, Lan D, Li X, Wang Y, Qi S, Liu Y (2023). Porphyromonas gingivalis is a risk factor for the development of nonalcoholic fatty liver disease via ferroptosis. Microbes Infect.

[82] Zhuge A, Li S, Yuan Y, Han S, Xia J, Wang Q (2023). Microbiota-induced lipid peroxidation impairs obeticholic acid-mediated antifibrotic effect towards nonalcoholic steatohepatitis in mice. Redox Biol.

[83] Liu S, Gao Z, He W, Wu Y, Liu J, Zhang S (2022). The gut microbiota metabolite glycochenodeoxycholate activates TFR-ACSL4-mediated ferroptosis to promote the development of environmental toxin-linked MAFLD. Free Radic Biol Med.

[84] Miao Z, Miao Z, Teng X, Xu S (2023). Melatonin alleviates lead-induced fatty liver in the common carps (Cyprinus carpio) via gut-liver axis. Environ Pollut.

[85] Huang S, Lin L, Wang S, Ding W, Zhang C, Shaukat A (2023). Total flavonoids of rhizoma drynariae mitigates aflatoxin B1-induced liver toxicity in chickens via microbiota-gut-liver axis interaction mechanisms. Antioxidants.

[86] Zeng Y, Wu R, Wang F, Li S, Li L, Li Y (2023). Liberation of daidzein by gut microbial β-galactosidase suppresses acetaminophen-induced hepatotoxicity in mice. Cell Host Microbe.

[87] Xu J, Tian H, Ji Y, Dong L, Liu Y, Wang Y (2023). Urolithin C reveals anti-NAFLD potential via AMPK-ferroptosis axis and modulating gut microbiota. Naunyn Schmiedebergs Arch Pharm.

[88] Deng F, Zhao B-C, Yang X, Lin Z-B, Sun Q-S, Wang Y-F (2021). The gut microbiota metabolite capsiate promotes Gpx4 expression by activating TRPV1 to inhibit intestinal ischemia reperfusion-induced ferroptosis. Gut Microbes.

[89] Liu Y, Jiao C, Zhang T, Li X, Li P, Lu M (2023). Early-life gut microbiota governs susceptibility to colitis via microbial-derived ether lipids. Res. (Wash. D. C.).

[90] Tang B, Zhu J, Fang S, Wang Y, Vinothkumar R, Li M (2021). Pharmacological inhibition of MELK restricts ferroptosis and the inflammatory response in colitis and colitis-propelled carcinogenesis. Free Radic Biol Med.

[91] Yang X, Sun X, Zhou F, Xiao S, Zhong L, Hu S (2023). Protocatechuic acid alleviates dextran-sulfate-sodium-induced ulcerative colitis in mice via the regulation of intestinal flora and ferroptosis. Molecules.

[92] Wu Y, Ran L, Yang Y, Gao X, Peng M, Liu S (2023). Deferasirox alleviates DSS-induced ulcerative colitis in mice by inhibiting ferroptosis and improving intestinal microbiota. Life Sci.

[93] Wang X, Li W, Dong Y, Zhang Y, Huo Q, Lu L (2023). Ferrostatin-1 mitigates ionizing radiation-induced intestinal injuries by inhibiting apoptosis and ferroptosis: an in vitro and in vivo study. Int J Radiat Biol.

[94] Cui W, Guo M, Liu D, Xiao P, Yang C, Huang H (2024). Gut microbial metabolite facilitates colorectal cancer development via ferroptosis inhibition. Nat Cell Biol.

[95] Liu S, Tang Y, Liu L, Yang L, Li P, Liu X (2022). Proteomic analysis reveals that ACSL4 activation during reflux esophagitis contributes to ferroptosis-mediated esophageal mucosal damage. Eur J Pharm.

[96] Huang C, Wu D, Zhang K, Khan FA, Pandupuspitasari NS, Wang Y (2022). Perfluorooctanoic acid alters the developmental trajectory of female germ cells and embryos in rodents and its potential mechanism. Ecotoxicol Environ Saf.

[97] Kong B, Fu H, Xiao Z, Zhou Y, Shuai W, Huang H (2022). Gut microbiota dysbiosis induced by a high-fat diet increases susceptibility to atrial fibrillation. Can J Cardiol.

[98] Zhang Y, Zhang P, Li Y (2022). Gut microbiota-mediated ferroptosis contributes to mercury exposure-induced brain injury in common carp. Metallomics.

[99] Guan Z, Jin X, Guan Z, Liu S, Tao K, Luo L (2023). The gut microbiota metabolite capsiate regulate SLC2A1 expression by targeting HIF-1α to inhibit knee osteoarthritis-induced ferroptosis. Aging Cell.

[100] Wang X, Zhang J, Wang S, Song Z, Sun H, Wu F (2023). Berberine modulates gut microbiota to attenuate cerebral ferroptosis induced by ischemia-reperfusion in mice. Eur J Pharm.

[101] Chen J, Guo P, Han M, Chen K, Qin J, Yang F (2023). Cognitive protection of sinomenine in type 2 diabetes mellitus through regulating the EGF/Nrf2/HO-1 signaling, the microbiota-gut-brain axis, and hippocampal neuron ferroptosis. Phytother Res.

[102] Geng Z, Nie X, Ling L, Li B, Liu P, Yuan L (2022). Electroacupuncture may inhibit oxidative stress of premature ovarian failure mice by regulating intestinal microbiota. Oxid. Med Cell Longev.

[103] Hu C, Liu M, Tang L, Liu H, Sun B, Chen L (2021). Probiotic intervention mitigates the metabolic disturbances of perfluorobutanesulfonate along the gut-liver axis of zebrafish. Chemosphere.

[104] Yue M, Wei J, Chen W, Hong D, Chen T, Fang X (2022). Neurotrophic role of the next-generation probiotic strain L. lactis MG1363-pMG36e-GLP-1 on Parkinson’s disease via inhibiting ferroptosis. Nutrients.

[105] Bridges R, Lutgen V, Lobner D, Baker DA (2012). Thinking outside the cleft to understand synaptic activity: contribution of the cystine-glutamate antiporter (System xc-) to normal and pathological glutamatergic signaling. Pharm Rev.

[106] Ferreira MJ, Rodrigues TA, Pedrosa AG, Silva AR, Vilarinho BG, Francisco T (2023). Glutathione and peroxisome redox homeostasis. Redox Biol.

[107] Xie Y, Kang R, Klionsky DJ, Tang D (2023). GPX4 in cell death, autophagy, and disease. Autophagy.

[108] Jin Z, Yang Y, Cao Y, Wen Q, Xi Y, Cheng J (2023). The gut metabolite 3-hydroxyphenylacetic acid rejuvenates spermatogenic dysfunction in aged mice through GPX4-mediated ferroptosis. Microbiome.

[109] Wen W, Xu Y, Qian W, Huang L, Gong J, Li Y (2023). PUFAs add fuel to Crohn’s disease-associated AIEC-induced enteritis by exacerbating intestinal epithelial lipid peroxidation. Gut Microbes.

[110] Chen H, Qian Y, Jiang C, Tang L, Yu J, Zhang L (2024). Butyrate ameliorated ferroptosis in ulcerative colitis through modulating Nrf2/GPX4 signal pathway and improving intestinal barrier. Biochim Biophys Acta Mol Basis Dis.

[111] Huang W, Chen H, He Q, Xie W, Peng Z, Ma Q (2023). Nobiletin protects against ferroptosis to alleviate sepsis-associated acute liver injury by modulating the gut microbiota. Food Funct.

[112] Doll S, Freitas FP, Shah R, Aldrovandi M, da Silva MC, Ingold I (2019). FSP1 is a glutathione-independent ferroptosis suppressor. Nature.

[113] Wang H, Ainiwaer A, Song Y, Qin L, Peng A, Bao H (2023). Perturbed gut microbiome and fecal and serum metabolomes are associated with chronic kidney disease severity. Microbiome.

[114] Fenn K, Strandwitz P, Stewart EJ, Dimise E, Rubin S, Gurubacharya S (2017). Quinones are growth factors for the human gut microbiota. Microbiome.

[115] Wang Y, Fan L, Huang J, Liang J, Wang X, Ren Y (2023). Evaluation of chemical composition, antioxidant activity, and gut microbiota associated with pumpkin juice fermented by Rhodobacter sphaeroides. Food Chem.

[116] Zheng J, Conrad M (2020). The metabolic underpinnings of ferroptosis. Cell Metab.

[117] Feng Q, Yang Y, Ren K, Qiao Y, Sun Z, Pan S (2023). Broadening horizons: the multifaceted functions of ferroptosis in kidney diseases. Int J Biol Sci.

[118] Belik J, Shifrin Y, Arning E, Bottiglieri T, Pan J, Daigneault MC (2017). Intestinal microbiota as a tetrahydrobiopterin exogenous source in hph-1 mice. Sci Rep.

[119] Dooling SW, Sgritta M, Wang I-C, Duque ALRF, Costa-Mattioli M (2022). The effect of limosilactobacillus reuteri on social behavior is independent of the adaptive immune system. mSystems.

[120] Wang Y, Tong Q, Ma S-R, Zhao Z-X, Pan L-B, Cong L (2021). Oral berberine improves brain dopa/dopamine levels to ameliorate Parkinson’s disease by regulating gut microbiota. Signal Transduct Target Ther.

[121] Nayak RR, Alexander M, Deshpande I, Stapleton-Gray K, Rimal B, Patterson AD (2021). Methotrexate impacts conserved pathways in diverse human gut bacteria leading to decreased host immune activation. Cell Host Microbe.

[122] Mishima E, Nakamura T, Zheng J, Zhang W, Mourão ASD, Sennhenn P (2023). DHODH inhibitors sensitize to ferroptosis by FSP1 inhibition. Nature.

[123] Molina-Vega M, Picón-César MJ, Gutiérrez-Repiso C, Fernández-Valero A, Lima-Rubio F, González-Romero S (2022). Metformin action over gut microbiota is related to weight and glycemic control in gestational diabetes mellitus: a randomized trial. Biomed Pharmacother.

[124] Yang Y, Chen T, Zhang X, Wang X (2021). Age-related functional changes of intestinal flora in rats. FEMS Microbiol Lett.

[125] Vaziri ND, Wong J, Pahl M, Piceno YM, Yuan J, DeSantis TZ (2013). Chronic kidney disease alters intestinal microbial flora. Kidney Int.

[126] Zhao J, Ning X, Liu B, Dong R, Bai M, Sun S (2021). Specific alterations in gut microbiota in patients with chronic kidney disease: an updated systematic review. Ren Fail.

[127] Wong J, Piceno YM, DeSantis TZ, Pahl M, Andersen GL, Vaziri ND (2014). Expansion of urease- and uricase-containing, indole- and p-cresol-forming and contraction of short-chain fatty acid-producing intestinal microbiota in ESRD. Am J Nephrol.

[128] Vaziri ND, Zhao Y-Y, Pahl MV (2016). Altered intestinal microbial flora and impaired epithelial barrier structure and function in CKD: the nature, mechanisms, consequences and potential treatment. Nephrol Dial Transpl.

[129] Wang X, Yang S, Li S, Zhao L, Hao Y, Qin J (2020). Aberrant gut microbiota alters host metabolome and impacts renal failure in humans and rodents. Gut.

[130] Yang T, Richards EM, Pepine CJ, Raizada MK (2018). The gut microbiota and the brain-gut-kidney axis in hypertension and chronic kidney disease. Nat Rev Nephrol.

[131] Anders H-J, Andersen K, Stecher B (2013). The intestinal microbiota, a leaky gut, and abnormal immunity in kidney disease. Kidney Int.

[132] Ramezani A, Raj DS (2014). The gut microbiome, kidney disease, and targeted interventions. J Am Soc Nephrol.

[133] Vaziri ND, Liu S-M, Lau WL, Khazaeli M, Nazertehrani S, Farzaneh SH (2014). High amylose resistant starch diet ameliorates oxidative stress, inflammation, and progression of chronic kidney disease. PLoS One.

[134] Sirich TL, Plummer NS, Gardner CD, Hostetter TH, Meyer TW (2014). Effect of increasing dietary fiber on plasma levels of colon-derived solutes in hemodialysis patients. Clin J Am Soc Nephrol.

[135] Koeth RA, Wang Z, Levison BS, Buffa JA, Org E, Sheehy BT (2013). Intestinal microbiota metabolism of L-carnitine, a nutrient in red meat, promotes atherosclerosis. Nat Med.

[136] Tang WHW, Wang Z, Levison BS, Koeth RA, Britt EB, Fu X (2013). Intestinal microbial metabolism of phosphatidylcholine and cardiovascular risk. N Engl J Med.

[137] Tang WHW, Wang Z, Kennedy DJ, Wu Y, Buffa JA, Agatisa-Boyle B (2015). Gut microbiota-dependent trimethylamine N-oxide (TMAO) pathway contributes to both development of renal insufficiency and mortality risk in chronic kidney disease. Circ. Res.

